# Dental Protective Association

**Published:** 1889-03

**Authors:** 


### Dental Protective Association.

This Organization, the Constitution of which was given in the January number of the REGISTER, is carrying forward its work with commendable energy and success, so far as the plan of operation is concerned. It should be borne in mind that "The International Tooth Crown Company" is an organization gotten up on the same plan and for the same purpose as the Dental Vulcanite Company of twenty years ago or more, and some of the same parties who were in that are also in this, and are now working with an accumulated experience, so that all the knowledge attained then, in that kind of work, will be made available now. There is this difference however between the operations of the former Company and those proposed by the present one, viz.: This Company proposes to buy up any and all Dental patents that are available, that can be used, as they used the Rubber Patent, and as they are using this, viz.: to make gain out of the Dentists on patents, that may be issued upon any instruments, materials, or modes of practice. If this was sought to be done with strictly valid patents, and only in a straight forward business-like manner and free from greed for gain, there would not be the same antagonism that is now entertained by dentists. The feeling in the profession now is, that as there is a strong organization formed and arrayed against them, one to whose power they would inevitably fall helpless, without support from each other; that the most simple, easy and efficient manner of accomplishing this is to place themselves so far as their interest in this matter is concerned, in the hands of "The Dental Protective Association of the United States." And it is to be hoped that this Association will have all needed support, both co-operative and financial. All information, that will aid and further the ends of Justice should be promptly given to the officers of this Association.

Money must be furnished for defense against unjust prosecutions, and to test the character of patents upon which demands for money may be made by the Crown Company.

Every member of the profession should come into this Association, and have his rights protected by proper adjudication.

It is to be hoped that every dentist will come into this Association and contribute his mite for protection.

Dr. J. N. Crouse, of 2231 Prairie Av., of Chicago, is Chairman of the Executive Committee of the Protective Association; he will answer all inquiries in regard to this matter, and receive and receipt for all contributions.

The membership fee is $10, which every one who wishes the aid that can be thus afforded him, should send at once.

